# Movement and dispersal of a habitat specialist in human-dominated landscapes: a case study of the red panda

**DOI:** 10.1186/s40462-021-00297-z

**Published:** 2021-12-14

**Authors:** Damber Bista, Greg S. Baxter, Nicholas J. Hudson, Sonam Tashi Lama, Janno Weerman, Peter John Murray

**Affiliations:** 1 School of Agriculture and Food Sciences (Wildlife Science Unit), The University of Queensland, Gatton, QLD 4343 Australia; 2 School of Sciences, University of Southern Queensland, West St, Darling Heights, QLD 4350 Australia; 3 Red Panda Network, Baluwatar, Kathmandu, 44600 Nepal; 4 Royal Rotterdam Zoological & Botanical Gardens, Postbus 532, 3000 AM Rotterdam, The Netherlands

**Keywords:** Activity pattern, Female-biased dispersal, Fragmentation effect, GPS telemetry, Human disturbances, Road effect

## Abstract

**Background:**

Habitat specialists living in human-dominated landscapes are likely to be affected by habitat fragmentation and human disturbances more than generalists. But there is a paucity of information on their response to such factors. We examined the effect of these factors on movement patterns of red pandas *Ailurus fulgens*, a habitat and diet specialist that inhabits the eastern Himalaya.

**Methods:**

We equipped 10 red pandas (six females, four males) with GPS collars and monitored them from September 2019 to March 2020 in Ilam, eastern Nepal. We collected habitat and disturbance data over four seasons. We considered geophysical covariates, anthropogenic factors and habitat fragmentation metrics, and employed linear -mixed models and logistic regression to evaluate the effect of those variables on movement patterns.

**Results:**

The median daily distance travelled by red pandas was 756 m. Males travelled nearly 1.5 times further than females (605 m). Males and sub-adults travelled more in the mating season while females showed no seasonal variation for their daily distance coverage. Red pandas were relatively more active during dawn and morning than the rest of the day, and they exhibited seasonal variation in distance coverage on the diel cycle. Both males and females appeared to be more active in the cub-rearing season, yet males were more active in the dawn in the birthing season. Two sub-adult females dispersed an average of 21 km starting their dispersal with the onset of the new moon following the winter solstice. The single subadult male did not disperse. Red pandas avoided roads, small-habitat patches and large unsuitable areas between habitat patches. Where connected habitat with high forest cover was scarce the animals moved more directly than when habitat was abundant.

**Conclusions:**

Our study indicates that this habitat specialist is vulnerable to human disturbances and habitat fragmentation. Habitat restoration through improving functional connectivity may be necessary to secure the long-term conservation of specialist species in a human-dominated landscape. Regulation of human activities should go in parallel to minimize disturbances during biologically crucial life phases. We recommend habitat zonation to limit human activities and avoid disturbances, especially livestock herding and road construction in core areas.

**Supplementary Information:**

The online version contains supplementary material available at 10.1186/s40462-021-00297-z.

## Introduction

Wildlife increasingly live in human-modified landscapes [1, 2]. Movement of animals through such landscapes is necessary to maintain ecosystem health and viability of local populations and metapopulations [3, 4]. An unwillingness to move through modified habitats makes habitat specialists especially vulnerable to human-induced fragmentation and disturbances [1, 3, 5], with implications for foraging, reproduction, and conspecific interactions [6–9]. Movement patterns usually vary across sex and age classes on a temporal scale [10–12]. An animal’s response to disturbances and risk may be more conspicuous in the presence of disturbances and risk, but it may differ based on the situation, proximity to the risk and landscape features where they alter their movement patterns to minimise the risk [13–15]. Furthermore, an animal’s responsiveness to disturbances may also be dependent on its species, age, sex, reproductive condition, nutritional condition and prior experience [3, 7, 16].

Variation in movement trajectory can be considered a proxy for response to risk and disturbances [3, 14]. Usually an animal’s movement path is direct, faster and shorter in areas with high risk and disturbance [3, 5, 14, 15]. Conversely, they follow a tortuous path and slowdown in high quality habitat [14]. The potential risk level also increases with an increase in the proportion of time spent in unsuitable areas [17]. Likewise animals modify their movement pattern across diel and seasonal cycles in response to disturbances [18]. Their dispersal pattern can also reveal some clues of the effects of disturbances [19, 20].

Usually the dispersal distance increases with home range and body size but other factors also influence this process [19]. For instance life history traits, social and environmental factors affect the dispersal distance which varies across species and even within members of the same species [19–21]. While this is true, the landscape structure strongly influences the dispersal ability of animals [14]. Animals residing in disturbed and fragmented habitats may experience high risk and avoid interactions with humans [20, 21]. Such movement is important in maintaining genetic diversity and minimizing inbreeding depression at the population level [20, 22]. However, in the absence of a particular type of habitat feature, as is more likely in fragmented habitats, habitat specialists usually have low dispersal capacity [23]. Despite the importance of dispersal information for conservation studying this process is challenging as young animals have to be observed. Furthermore it becomes more complicated in endangered species as only a few individuals are available for study [24].

**Fig. 1:**
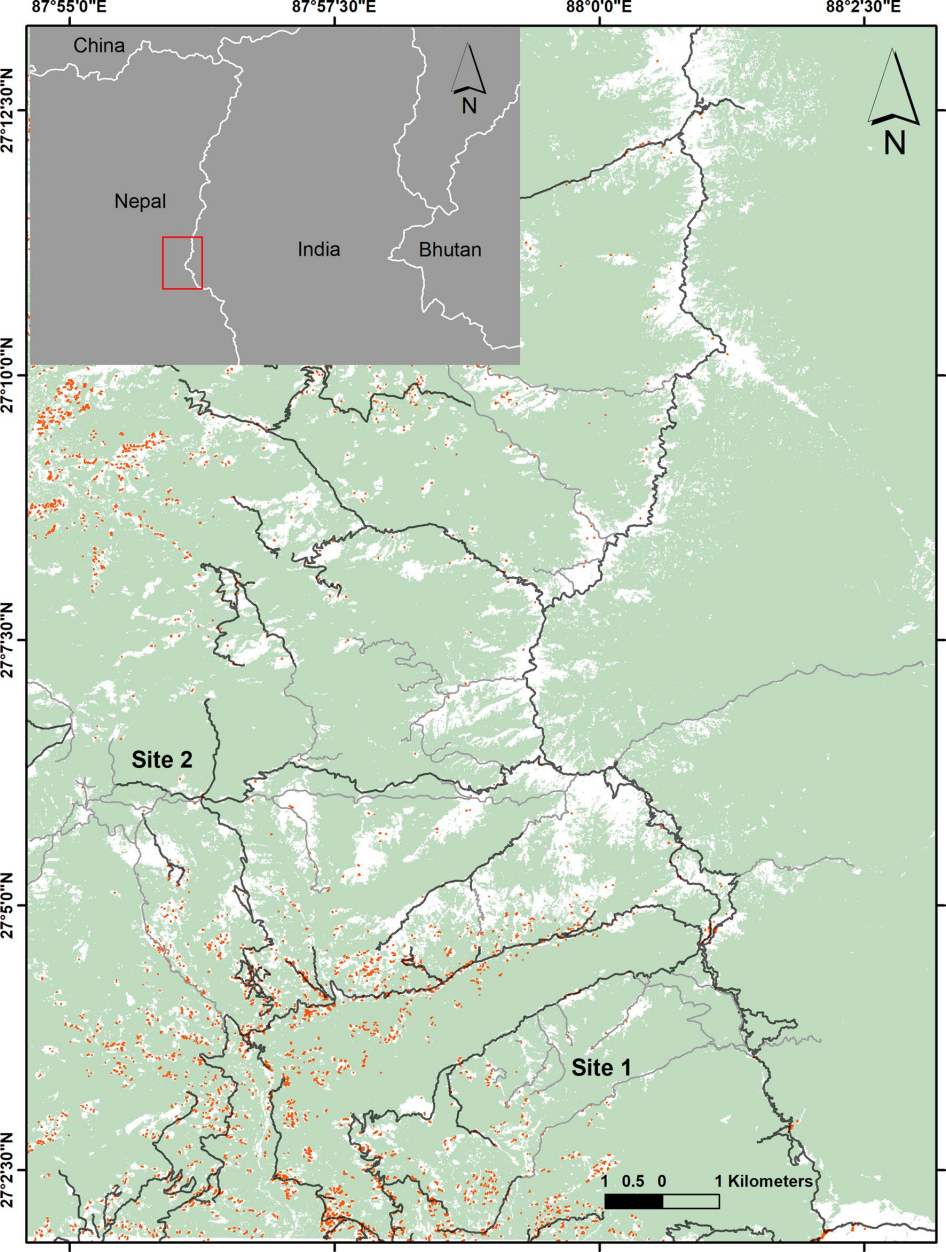
The inset shows the study area located in eastern Nepal bordering India in the east. The study was carried out in Ilam and Panchthar districts in eastern Nepal. The elevation of study area ranges from 1500 to 3636 m. Black and grey lines represent road and human tracks respectively, while human habitations are shown as orange dots. Light green shading shows forest and white represents non-forest areas. We collared 10 animals including four males and six females in two sites: Site 1 (7 animals: 3 males, 4 females) and Site 2 (3 animals: 1 male, 2 females). It is clear from this figure that some areas of the study site are relatively close to human disturbance whereas others are relatively remote

A recent study on an arboreal specialist folivore, the koala *Phascolarctos cinereus*, reported adverse effects of habitat fragmentation on movement patterns [5]. Nevertheless there is a lack of information on how arboreal habitat specialists inhabiting mountain habitat deal with disturbances and habitat fragmentation. Therefore we aimed to study the effects of human disturbances on the montane, red panda *Ailurus fulgens,* in a human-dominated landscape. This endangered species is a habitat and diet specialist of the Eastern Himalaya [25, 26]. In our study area it lives in an environment composed of a mixed matrix of pristine and disturbed habitat patches (Fig. 1). High resolution GPS tracking of red panda through this well characterised landscape matrix allowed us to estimate the response of the red panda to both natural factors and anthropogenic disturbances in detail that has not previously been possible.

The red panda has a restricted range in temperate forests with abundant bamboo within an elevation from 2300 to 4000 m with sporadic records beyond this range [26]. Red pandas also represent a group of herbivorous members of the order Carnivora having a specialized diet with poor nutrient content [27–29]. In order to obtain their energy requirements red pandas spend long hours foraging and remain less active outside feeding hours [30]. For this reason their active phases are interspersed with short resting periods [10, 30, 31]. This mammal is facing acute threats due to habitat loss and fragmentation [26], and poaching [32]. Over the red panda’s range land cover and usage is rapidly changing due to increasing development and human population growth [33]. Their global population in the wild has declined by nearly 50% in three generations i.e. less than 20 years [26]. This issue has raised the need for a better understanding of the movement ecology of red pandas in disturbed habitat. Previous studies have reported the avoidance of interactions with humans [34–36] and an increase in red panda home range size in areas with low forest cover [36]. However, none of those studies [10, 31, 37] attempted to investigate red panda’s responses to disturbances and habitat fragmentation from a movement perspective.

We tested four *a-priori* hypotheses that red pandas: (1) have movement patterns that vary across sex and age classes on a seasonal scale, (2) are active throughout the diel cycle with only short passive periods, (3) move faster and more directly in risky and fragmented habitat, and (4) exhibit risk avoidance behaviour when dispersing.

## Methods

### Study area

This study was carried out in Ilam and Panchthar district, eastern Nepal (Fig. 1). The study area borders with Singalila National Park in India to the east. This area has sub-tropical and temperate climate. The mean annual temperature of the study area was 13.1 °C (SD 6.78, range − 1 to 28.9 °C; Additional file 1: Table S1) with annual precipitation of 2590 mm [38]. This area harbours many other mammals including marbled cat *Pardofelis marmorata*, Asiatic golden cat *Catopuma temminckii*, leopard cat *Prionailurus bengalensis,* leopard *Panthera pardus,* clouded leopard *Neofelis nebulosa,* tiger *Panthera tigris,* Northern red muntjac *Muntiacus vaginalis*, wild boar *Sus scrofa,* Himalayan goral *Naemorhedus goral,* Himalayan serow *Capricornis thar,* Assam macaque *Macaca assamensis,* yellow-throated marten *Martes flavigula,* and Himalayan black bear *Ursus thibetanus* [39, 40]. The study area was ideal to study red panda movement patterns in a human-modified landscape as the component patches were surrounded to varying extents by human settlements with roads, human-walking tracks and livestock grazing activities present throughout the year. There were more than 15 settlements with a total population of nearly 700 people living around the selected site [41]. The study area had a road density of 3.84 ± 3.7 km/km^2^.

### Data collection

We captured 10 red pandas (six females and four males) using cage traps and equipped them with GPS satellite collars (LiteTrack Iridium 150 TRD) following a standard operating procedure (see detail in [42]). Of these collared animals, seven were adults (four females, three males) and three were sub-adults (two females, one male). We monitored them from September 2019 to December 2020. The GPS collars were set with one fix every 2 h, transferred remotely. Our GPS telemetry data had two issues: missing location fixes, and imprecise locations of successfully acquired fixes. Therefore we omitted data with unsuccessful GPS fixes. We also omitted imprecise data with the dilution of precision > 5 [43]. Further, we retained only those fixes having a minimum of 2-h interval of each animal. Finally, we retained only locations at possible elevations for our study area between 1500 and 3636 m. We tested the collar for errors in the field and found errors up to 25 m. Hence, we considered 25 m as the threshold of telemetry errors in further analyses.

We also carried out camera trapping to quantify human, livestock, free-ranging dog and vehicular traffic. We deployed 34 trail cameras (Bushnell 20/24MP Trophy Cam HD No-Glow, X-change Color Model 1279) along roads, human trails and forest areas and fastened each camera to a tree at 40 cm above the ground. We placed these cameras at a minimum of 250 m apart. We used the program Camera Base 1.7 to sort the camera data [44]. We considered image captures of an individual taken 30 min apart as an independent event.

### Data analyses

#### Movement and dispersal

We estimated home range size as weighted Autocorrelated Kernel Density estimation at 95% isopleth in the ctmm package [45]. Initially we calculated step length (Table 1) and time lag using the move package [46]. Then we corrected telemetry errors, calculated the time lag between successive GPS fixes and retained those fixes with a 2-h time lag to avoid duplication in distance aggregation. We calculated the moon fraction for each location and considered the value from 0 to 1 representing new moon and full moon respectively [47]. We estimated movements during four periods of the diel time: dawn, day, dusk and night, and moon fraction using the suncalc package [47]. We considered four seasons based on red panda biology: premating (November–December), mating (January–March), gestation and birthing (hereafter birthing, April–July), and cub-rearing (August–October).

**Table 1:** Description of variables used in movement analyses

Variables	Description
Demographic variables	
Sex	Male, female
Age	Adult, sub-adult
Temporal variables	
Season	Mating (January–March), Birthing (April–July), Cub-rearing (August–October), Premating (November–December)
Diel time	Dawn (period between astronomical dawn when the sun is at 18° below the horizon and golden hour after sunrise), Dusk (period between golden hour before sunset and astronomical dusk), Day (period between two golden hours after sunrise and before sunset), Night (period between astronomical dusk and astronomical dawn)
Movement metrics	
Step length	The Euclidean distance (m) between two consecutive GPS fixes of an animal that were recorded at an interval of 2 h
Distance	Refers to the sum of step length distances (m) covered by an individual in 24 h. It refers to the daily distance unless otherwise specified
Dispersal distance	The topographic distance (km) following contours between the natal and the new home range. It refers to the total distance covered by a disperser unless otherwise specified
Straightness index	The ratio of the square root of net square displacement, i.e. the square of the Euclidean distance between two points, divided by the sum of the step lengths of the movement trajectory of each red panda for each season [98]. These two points were the start and end points of the movement trajectory. Values range from 0 to 1 relating to increasing straightness with higher values
Geo-physical variables	
Elevation (Elev)	Elevation of red panda presence points (m). Source: Shuttle Radar Topography Mission (SRTM, 1 arc-second) Global Digital Elevation Model (DEM)—https://earthexplorer.usgs.gov/
Topographic Position Index (TPI)	Topographic Position Index measures elevation difference. Values ranged between − 9 and 10 with higher values being mountain ridges and lower values being mountain valleys. Source: SRTM, 1 arc-second, DEM
Slope	Slope of red panda presence points (°). Source: SRTM, 1 arc-second, DEM
Fragmentation metrics within home ranges	
Habitat patch	Refers to the set of neighbouring cells belonging to same land cover type, i.e., forest cover
Fractal Dimension Index (FRAC)	Describes the shape complexity of each habitat patch based on perimeter-area relationships. Values range between 1 and 2 with very simple perimeters close to 1 and highly convoluted complex shape towards 2 [52]. Habitat patches due to natural causes have more complex and irregular shapes while human-induced patches have regular shapes
Patch density (PD)	Is the number of forest habitat patches in the home range of an animal divided by the home range area (number of patches/ha)
Edge density (ED)	Sum of total length of edge of forest habitat patches within an animal’s home range (m/ha)
Proportion of land cover (PLAND)	We included two land cover type: forest and non-forest. PLAND represents the proportion of forest cover in a home range (%)
Patch area (AREA)	The area (ha) of each habitat patch
Connectance Index (CONNECT)	Refers to the percentage of the maximum possible connection among the forest habitat patches within the Euclidean distance of 50 m, see details in [52]. A zero value refers to a single patch or no connection between any patches, while 100 means there is connection between all patches [52]
Clumpiness Index (CLUMPY)	Refers to the distribution pattern of forest habitat patches. It ranges from − 1 to 1. A zero value refers to random distribution of patches, and values close to − 1 and + 1 show increasing dispersal and increasing clumpiness of patches respectively, see details in [52]. The index will be 1 for single patch in a landscape
Euclidean Nearest Neighbour Index (ENN)	Refers to the shortest straight-line edge-to-edge distance between two forest habitat patches (m)
Disturbance variables	
Distance to road (Road_dist)	Euclidean distance between the red panda presence points and the nearest road (m). Source: https://www.openstreetmap.org/
Distance to human-walking track (Trac_dist)	Euclidean distance between the red panda presence points and the nearest human-walking trail (m): Source: https://www.openstreetmap.org/
Distance to cattle station (Catt_dist)	Euclidean distance between the red panda presence points and the nearest cattle station (m)

Animal movement is directed by the topography in their habitat [48]. We therefore considered the topographic distances following contours between natal and new home ranges as the dispersal distances (Table 1) which are longer than the straight-line distances between two points [48]. We calculated dispersal distances with the TopoDistance package [49]. To do this we obtained 30 m resolution elevation data from Shuttle Radar Topography Mission [50], and extracted elevation, slope and Topographic Position Index (TPI, Table 1).

#### Effect of disturbance and fragmentation

We considered the Euclidean distance between a red panda location and: road, settlement, human-walking track, and cattle station as indicators of human disturbance (Table 1) and located these features in ArcMap 10.8. Each herder had at least two cattle stations where they move seasonally with their livestock. We accessed Sentinal-2A satellite images of the study area between 15 October 2019 and 13 January 2020 at 10 m spatial resolution, reclassified into two land-cover types: forest and non-forest [51], and extracted the land cover area within seasonal home ranges of each animal.

We considered patch as well as class-level metrics (Table 1) to analyse the habitat fragmentation effects in the home range of an animal. Patch area (AREA) and the proportion of land cover availability (PLAND) were used to quantify the land cover type in home ranges. Shape describes complexity of patches and reveals the causes behind the fragmentation [52]. Patches due to natural causes have more complex and irregular shapes while the human-induced patches have regular shapes [52]. We used the Fractal Dimension Index (FRAC) to measure shape complexity. Aggregation is a key aspect of landscape ecology which refers to the degree to which patches are spatially aggregated [53]. Therefore we chose the Connectance Index (CONNECT), Clumpiness Index (CLUMPY), Patch Density (PD), and Euclidean Nearest Neighbour Index (ENN) metrics at a landscape level to measure the aggregation of any given habitat [52–54]. We estimated these fragmentation metrics using Fragstat v4.2.1 [55]. We used the straightness index (Table 1) to measure the shape of red panda movement paths to quantify disturbances due to human activities and fragmentation [56].

### Statistical analyses

Initially we examined multicollinearity of predictors and retained only those with a variation inflation factor smaller than 5 [57]. We standardised continuous predictors by centering around the mean with a unit standard deviation [58]. We fitted four Linear Mixed Models (LMM) in lme4 package [59] and included individual identity (i.e., unique code of each study animal) as a random term.

- Variation in distance coverage with daily distance as the response variable and sex, age and season as fixed factors. An animal’s behaviour varies with age and sex due to changes in the physical environment of habitat and biological requirements across seasons [11]. We therefore included the interaction terms season by age and season by sex.
- Variation in activity pattern with step length as the response variable and sex, age, season and diel time as fixed factors. In addition to the additive effect of these four predictors, we included the interaction terms season by sex, season by diel time, and sex by diel time in the global model.
- Response to disturbances at patch level using step length as the response variable, and eight fixed factors: distance to cattle station, road and track, AREA, ENN, FRAC, season and diel time. Herders move their cattle to low elevation during the winter and reverse their movement in the summer. We hypothesised that red pandas respond to such seasonal movement of cattle by avoiding those sites or partition their movement pattern to minimize disturbances. We therefore included the interaction terms distance to cattle stations by season and distance to cattle stations by diel time in the full model with step length as the response variable.
- Movement pattern at landscape level using the straightness index as the response variable, and PLAND, AREA, CLUMPY and CONNECT as fixed factors.

We examined the difference in distance travelled (sum of step length distances) across the diel time using Kruskal–Wallis rank sum test and performed post-hoc Dunn test to compare pair-wise differences. We considered step length as a proxy of activity pattern. Then we plotted the activity pattern across the diel time.

The time-series plot can be used to visualize the distance between a pair of animals in any given time [60]. We plotted the time-series plot to assess the distance between mothers and their dependent cubs. Furthermore, we fragmented their movement trajectory to identify dispersal and non-dispersal phases with the segclust2d package [60]. After identifying the dispersal and non-dispersal phases, we tested differences in daily distance travelled using the Wilcoxon-signed-rank test. Then we evaluated the effect of elevation, slope, TPI, step length, and distance to the nearest cattle station and walking track. We used the binary response (dispersal phase and non-dispersal phase) as the response variable in the generalized linear modelling with the binomial family and logit link function to evaluate the effects of variables during the dispersal phase.

We used model selection to test all combinations of the predictor variables; model selection was based on Akaike's information criterion (AIC) using the MuMIn package [61]. We selected the model with the smallest AIC [62] but averaged models if more than one model was within AIC value of 4 [63].

## Results

We recorded 13,749 telemetry locations of 10 red pandas between 22 September 2019 and 15 December 2020. We retained 6947 locations within the 2-hour time lag after removing outliers and erroneous locations. Disturbance variables and fragmentation metrics were interlinked at patch level (Additional file 1: Fig. S1), and they had skewed distributions (Additional file 1: Fig. S2a). Distances between the disturbances and randomly generated points also exhibited similar patterns (Additional file 1: Fig. S2b). We estimated median annual home range of red pandas as 1.41 km^2^ (male 1.73 km^2^, female 0.94 km^2^, see detail in [64]). We found the median (interquartile range [IQR]) forest cover as 92.8% (90.8–98.1%) with PD of 5 (2.8–7.1) within each animal’s home range. Median area of these forest patches was 19.2 ha (12.5–34.4 ha). The median values of clumpiness and connectance indices were 0.7 (0.5–0.7) and 66.7 (29.3–100) respectively. Median FRAC of forest patches was 1 (1–1.1) while the median Euclidean distance between two neighbouring forest patches was 22 m (20–30 m) with maximum distance up to 125 m. Median distance between red panda presence points and disturbance sources varied: cattle stations (1415 m [1224–1681 m]), human tracks (134 m [60–451 m]), road (309 m [212–616 m]) and settlement (573 m [400–781 m]).

### Daily distance

**Fig. 2:**
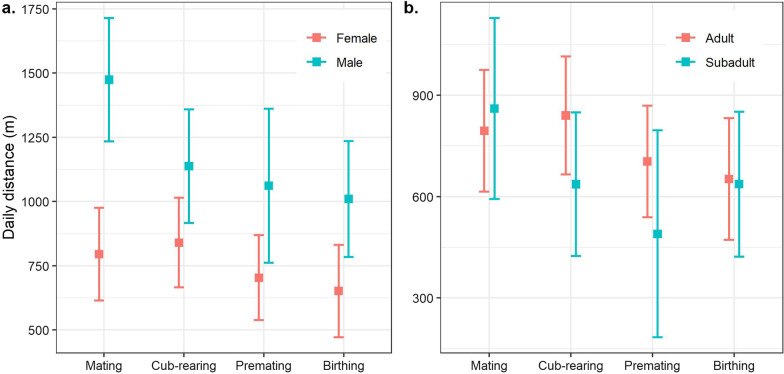
Predicted daily distance travelled by red pandas during the four seasons. **a** The blue line represents the distance travelled by males (n = 4), and the red line represents females (n = 6). **b** The blue line is for subadults (n = 3) and the red line is for adults (n = 7). The square box represents the predicted distance while error bars on both sides show the 95% CI

The median (IQR) daily distance travelled by red pandas was 756 m IQR 518–1167 m [males: 953 m (660–1473 m), females: 605 m (419–841 m)]. Males travelled nearly double the distance (mean 1474 m) of females (mean 795 m) in the mating season (*β* = 679.14, *p* < 0.001, Fig. 2a, Additional file 1: Tables S2 and S3). Males were less active during cub-rearing (*β* =  − 381.5, *p* < 0.01), birthing (*β* =  − 321.1, *p* < 0.03) and premating (*β* =  − 321.1, *p* = 0.06) seasons than in the mating season. There was no difference for the distances females travelled over the seasons although they travelled marginally less in the birthing season (*β* =  − 143.16, *p* = 0.09, Fig. 2a). Subadults covered longer distances (mean 861 m) than adults (mean 795 m) in the mating season and travelled less than adults in other seasons (Fig. 2b). However, these differences were not significant (Additional file 1: Table S3). We recorded the maximum daily distance travelled as 5300 m by an adult male during the premating season.

### Movement in diel cycle

**Fig. 3:**
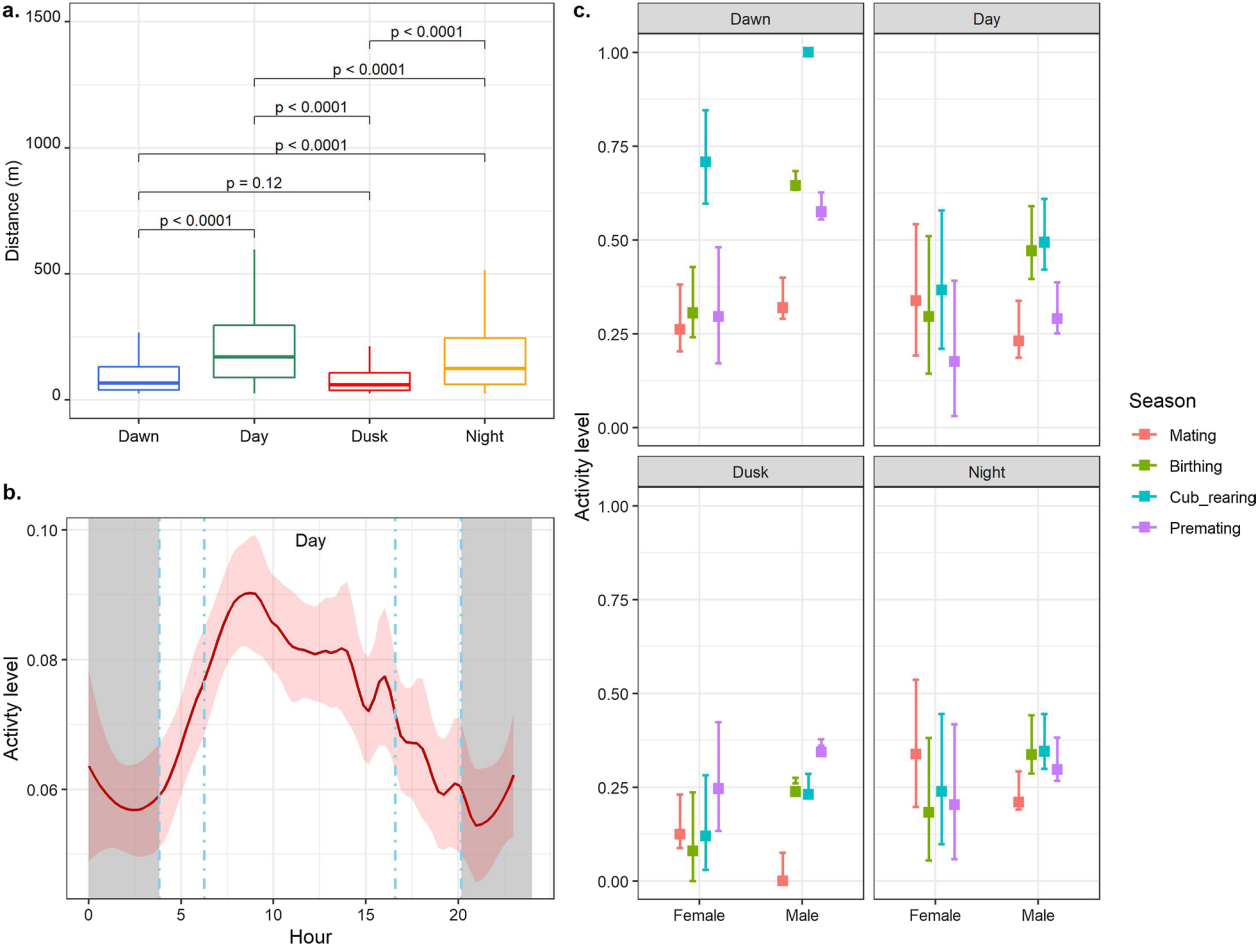
Distance travelled and activity levels across the diel cycle. **a** Statistical summary of the distance travelled across the diel time. Estimated distance is based on the raw data. The Kruskal–Wallis rank-sum test showed significant differences in distance travelled during these different times (*p* < 0.001). Further, the post-hoc Dunn test revealed significant differences between the distance travelled between five pairs except dawn-dusk. **b** Activity patterns of red pandas across the diel cycle. This is based on annual raw data. We took step length as a proxy of activity which is scaled between 0 to 1. The area between two sky-blue lines represents dawn and dusk, the grey area shows night and the wide area between two sky-blue lines is day. The thin ribbon around the line shows the 95% CI. **c** Predicted activity levels of males and females during the diel cycle across seasons. Step length was considered as proxy of activity level. The square box and error bars represent the predicted activity level and 95% CI respectively. Each colour depicts a season (see legend). Predicted values and confidence intervals are scaled between 0 and 1

Distance travelled across the diel time varied significantly (Kruskal–Wallis rank sum test = 198.7, *df* = 3, *p* = 0). The post-hoc Dunn test showed that red pandas covered longer distances in the day and night than in the dawn and travelled the least distance at dusk (Fig. 3a). They exhibited a uni-modal pattern but with minor peaks in the afternoon, evening and night (Fig. 3b). They were more active from dawn until 3–4 h after sunrise. Their activity levels gradually decreased but fluctuated until mid-night. We observed no variation in activity patterns between adults and subadults although it differed across sex classes. The seasonal activity pattern of males and females differed across the diel time (Additional file 1: Tables S4 and S5, Fig. 3c). Female’s activity level was higher at dawn while rearing their cubs than during the mating season (*β* = 14.3, *p* < 0.006). They remained more active at day (*β* =  − 13.4, *p* < 0.02) and less active in the dusk (*β* =  − 14.5, *p* < 0.05) and night (*β* =  − 17.5, *p* < 0.003) while rearing their cubs than in the mating season (Additional file 1: Table S5, Fig. 3c). Males were also more active at dawn of the cub-rearing (*β* = 7.7, *p* < 0.06, Fig. 3c) and birthing (*β* = 9.25, *p* < 0.03, Additional file 1: Table S5, Fig. 3c) seasons than in the mating season.

### Dispersal

We had only two female sub-adult red pandas collared but both of them separated from their mothers with the onset of the new moon between 30 January and 7 February, when they were seven to eight months old (Additional file 1: Fig. S3). Age estimation was based on the birthing records of two adult females in early July 2020. One disperser left her natal home three weeks after separation from her mother, and the other after five weeks. The former reached a new range in 42 days and the latter took 26 days. Both sub-adults spent another 42 to 44 days exploring their new range before establishing their territory. Clustering of movement trajectories further aided in demarcation of dispersal and non-dispersal phases (Additional file 1: Fig. S4). We also observed one more (uncollared) cub of a collared mother living alone in the natal site on 26 February 2020. Another three uncollared cubs of two collared mothers also dispersed before mid-February in the following year. The two sub-adult females spent nearly three months dispersing before settling in a new home. Their dispersal distance was 17.9 and 24.1 km respectively (Additional file 1: Fig. S5). The median daily distance travelled during dispersal was 584 m (IQR 332–1059 m) which was significantly more than during the non-dispersal phase: 405 m (IQR 259–582 m; Wilcoxon-signed-rank test, *Z* = 8.1, *p* < 0.001). All dispersing animals had 6 to 7 stopover sites where they spent 1 to 4 days during this journey.

The best-fit model to explain the variation between dispersal and non-dispersal phases included distance to human-walking tracks, distance to cattle stations, elevation, TPI, step length and slope (Table 2). Dispersers exhibited affinity for low elevations and avoided mountain ridges and areas close to cattle stations and human tracks (Table 3).

**Table 2:** Models describing variables affecting the dispersal phase of red pandas (with dispersal phase as reference)

Models^*#*^	df	AIC	ΔAIC	weight
Step_leng + TPI + Elev + Catt_dist + Trac-dis	6	262.2	0	0.67
Step_leng + TPI + Slope + Elev + Catt_dist + Trac_dist	7	264.2	2.01	0.24
TPI + Elev + Catt_dist + Trac_dist	5	268.2	5.97	0.03
Step_leng + Elev + Catt_dist + Trac_dist	5	268.5	6.28	0.03

We included step length, Topographic Position Index, aspect, slope, elevation, distance to road, and distance to cattle stations as predictors. First four top models resulting from model selection based on AIC are shown. Models with ΔAIC < 4 were averaged

^*#*^Step_leng: step length, TPI: Topographic Position Index, Catt_dist: distance to cattle station, Trac_dist: distance to walking tracks

**Table 3:** Effects of predictors on dispersal phase. Estimates are based on the averaged model with ΔAIC < 4 (see Table 2)*

Variables^*#*^	Estimate	SE	z-value	*p*
Intercept	**−** 24.94	6.33	3.93	0.00
**Catt_dist**	**−** **0.01**	**0.00**	**5.43**	**0.00**
**Trac_dist**	**−** **0.002**	**0.00**	**7.13**	**0.00**
**Elev**	**0.011**	**0.00**	**4.41**	**0.00**
Step_leng	0.001	0.00	1.25	0.21
**TPI**	**−** **0.19**	**0.07**	**2.77**	**0.01**
**Slope**	0.001	0.02	0.09	0.93

Significant estimates are highlighted in bold

*Tjur’s *R*^2^ = 0.31, ^#^Catt_dist: distance to cattle station, Trac_dist: distance to walking tracks, Elev: elevation, Step_leng: step length, TPI: Topographic Position Index

### Effect of disturbance and fragmentation

**Fig. 4:**
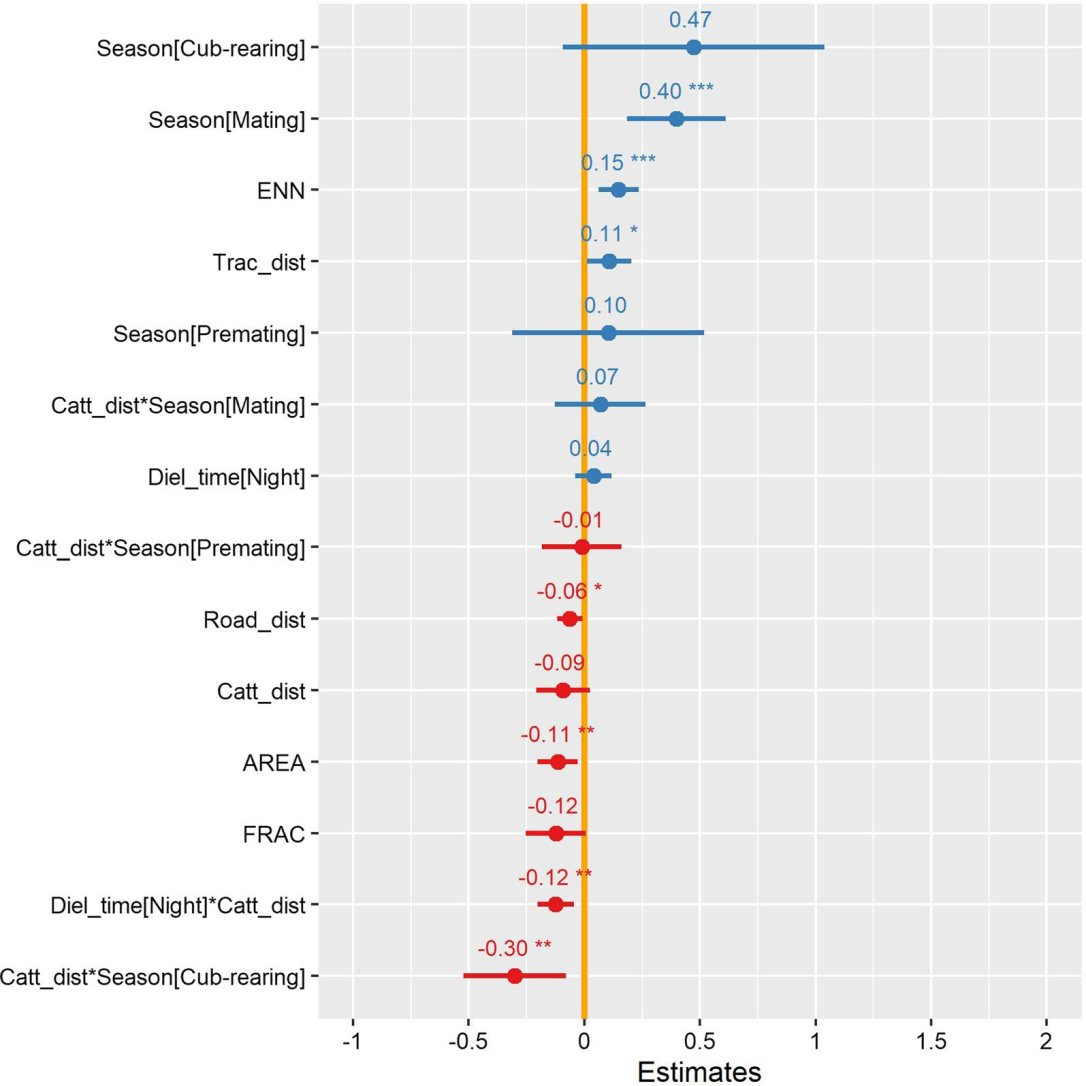
Parameter estimates of variables affecting red panda step length. Estimates are based on averaged model from the set of top models with ΔAIC < 4 (see Additional file 1: Table S6). The yellow line represents the zero effect. Significant responses are highlighted with stars. The x-axis depicts estimates while the y-axis represents variables. Asterisks indicate significance level: * = 0.05, ** = 0.005, *** = 0.0005

The averaged model showed a clear relationship between step lengths, human disturbance and fragmentation metrics (Fig. 4, Additional file 1: Table S6). Red pandas moved with a significantly longer step length while approaching roads (*β* =  − 0.06, *p* < 0.02). In contrast they slowed down in areas close to human-walking tracks (*β* = 0.11, *p* < 0.02). Their step length during the night was longer than during the day (*β* = 0.04) although it was not significant. Red pandas moved more slowly when moving away from cattle stations in the night than they did in the day (*β* =  − 0.12, *p* < 0.002). The effect of cattle distance was conspicuous in the cub-rearing season as red pandas’ step lengths were lower when far from cattle stations (*β* =  − 0.3, *p* < 0.007). Apart from this, their response to disturbances did not vary across seasons although their step lengths were longer during the day in the mating season than during the birthing season (*β* = 0.39, *p* < 0.001). Their step lengths were also longer in small-sized habitat patches (*β* =  − 0.11, *p* < 0.001), and they moved faster between two habitat patches when the inter-patch distance was high (*β* = 0.15, *p* < 0.001).

**Fig. 5:**
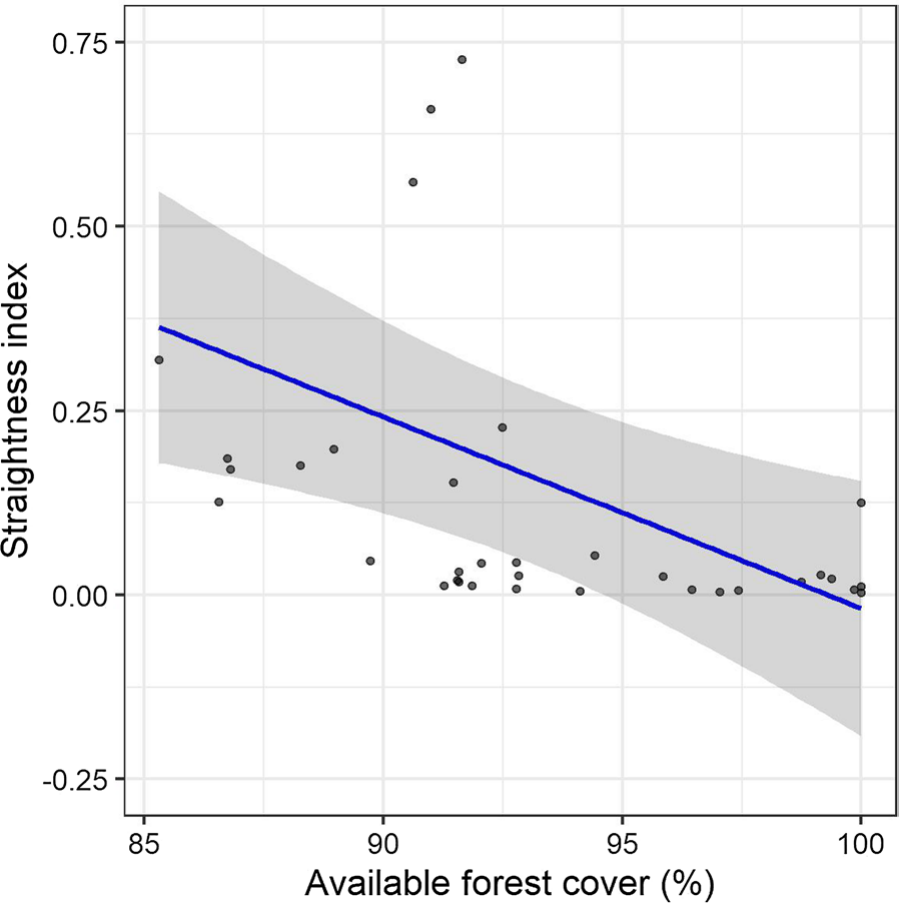
The available forest cover (PLAND) affected straightness of movement trajectory. This plot is based on the prediction of linear-mixed model (see Table 4). The PLAND and straightness index are shown in x and y axes respectively. The value of straightness index ranges from 0 to 1 relating to increasing straightness with higher values

PLAND and CLUMPY were the best explanatory predictors of the straightness of movement path in the averaged model (Table 4), but only PLAND had a significant influence (*β* =  − 0.03, *p* < 0.001, Fig. 5).

**Table 4:** Models describing the straightness index as a function of PLAND, AREA, CLUMPY, CONNECT*

Models^*#*^	df	AICc	ΔAICc	Weight
Null model	3	− 9.4	0	0.48
PLAND	4	− 8.3	1.12	0.27
CLUMPY	4	− 7.5	1.89	0.19
CLUMPY + PLAND	5	− 5.2	4.23	0.06

First top four models resulting from model selection based on corrected Akaike’s Information Criterion (AICc). Models with ΔAICc < 4 were retained for model averaging

*Marginal *R*^2^ = 0.24, conditional *R*^2^ = 0.73, ^*#*^PLAND: proportion of land cover, CLUMPY: Clumpiness Index

## Discussion

Firstly we conclude that this investigation was conducted in habitat that was clearly disturbed by human activities. All disturbance variables had right skewed distributions (Additional file 1: Fig. S2a). Distances between the disturbances and randomly generated points also exhibited similar patterns (Additional file 1: Fig. S2b). Since the randomly placed points also showed the same skewed distribution, we suggest that it was not possible to move large distances from these human disturbances because the habitat patch size was small. In other words red pandas had no choice but to stay close to human disturbances because they could not get far away from disturbance without exiting the habitat patch. Here we showed that red pandas avoided areas close to roads, human tracks, and cattle stations. They also avoided small, isolated habitat patches and areas with low forest cover. Red pandas responded to habitat fragmentation at both patch and landscape levels. Overall, they travelled (1) faster during the night than in the day; (2) long-distances at night while the day was mostly for foraging; and (3) faster in smaller patches and their speed was higher in unsuitable landscape matrix between neighbouring patches. There was also seasonal variation in daily distance travelled and activity pattern across the diel cycle.

### Daily distance

Consistent with our a priori hypothesis, red pandas exhibited seasonal variation across sex and age classes on a seasonal scale. Male red pandas travelled longer distances than females which corroborates previous studies [10, 31, 65]. This trait is common in territorial males in mammals who travel longer distances to maintain their territory [66]. Their relatively longer distance coverage during the mating season could be attributed to maintaining territory and finding mating partners [67]. We found red panda males meeting up to three females during a mating season. However, average daily distance travelled by red pandas in this study was found to be nearly double that identified in previous research [10, 31, 65]. Use of GPS telemetry leading to higher sampling resolution may have captured this variation as the previous studies were based on VHF collars.

Females usually travel shorter distances during gestation and birthing seasons than in other seasons. Pregnant females need more energy and do not expend energy by travelling longer distances, rather they stay in a small area before parturition [68]. Further, red panda gestation overlaps with the pre-monsoon and early-monsoon season when an abundance of rainfall increases food availability [29]. A female’s calorific requirement is higher during lactation [69] which lasts beyond the rainy season when food availability starts to dwindle forcing them to travel more to find enough food to increase energy intake. Previous studies have also reported less distance coverage during gestation and birthing seasons which was relatively higher in the cub-rearing season [10, 31, 65]. These movement patterns suggest that season-specific energetic requirements drive the movement pattern in females.

### Activity pattern in diel cycle

Our observation supports the hypothesis that red pandas are active throughout the diel cycle with only short passive periods. They were relatively more active during the dawn and morning hours with slight fluctuations throughout the diel cycle. This observation is in line with other reports [10, 37, 70], but it contradicts the crepuscular pattern reported elsewhere [10, 70]. Our observation is similar to some other specialists having a specialized bamboo diet, such as the giant panda *Ailuropoda melanoleuca* [12], southern bamboo lemur *Hapalemur meridionalis* [71] and bale monkey *Chlorocebus djamdjamensis* [72]. Such a prolonged activity pattern could be attributed to their trade off to obtain optimal energy from the nutritionally poor diet [12, 30]. Animals relying on poor-quality diet have to spend more time foraging in degraded habitat which may affect their welfare in the long run. These include species feeding on bamboo, such as the golden monkey *Cercopithecus mitis kandti* [73], mountain gorilla *Gorilla beringei beringei* [74], and eucalyptus, such as the koala [75].

The energetic cost, resource availability and predation risk vary across seasons [76–78], which have a direct effect on animal’s activity patterns. This effect was obvious in our study as we observed red pandas to be more active during the cub-rearing season with a nearly uniform pattern in other seasons. Their activity patterns also varied at an individual level: random variance = 84 (SD 9.2). Such variation at an individual level could be related to an individual’s adaptive traits in response to extrinsic factors [10, 79]. However, the narrow confidence interval of activity level of males suggest that they remain more active throughout the year unlike females (Fig. 3c).

### Dispersal

Despite a small sample size, our study generates a hypothesis for future tracking studies on dispersal of red pandas which here exhibited female-biased dispersal. The only two studied sub-adult females dispersed, while the single sub-adult male did not. The finding of female-biased dispersal is consistent with Hu et al. [25] who’s report was based on genetic evidence. However, the small sample size warrants caution at this stage of knowledge. A similar pattern of dispersal is also evident in the giant panda, another species with a similar diet [80], and many primates [81]. We recorded 21 km mean dispersal which is relatively large for arboreal mammals and herbivores of this size [19, 22]. For instance, average dispersal distance of the koala, a solitary and arboreal mammal with a folivorous diet is 3.5 km (range 0.3–10.6 km) [82]. Roads and similar other linear features may act as barriers for dispersal as one individual appeared to have shortened its dispersal distance after encountering a road along her path. This observation underpins the need for landscape-level planning for red panda conservation.

The cost of dispersal varies with disturbances and photoperiod [83, 84]. Red pandas moved more slowly on luminous nights than on dark nights, which could be due to increased vigilance to avoid predators in brighter moon light. This observation suggests that artificial light could affect these key ecological processes of red pandas and other lunar-phobic species [85–87].

In our study, red pandas travelled longer distances during the dispersal phase which overlapped with the mating season. Further, they stayed at low elevations and avoided cattle stations and human trails, which suggests an ability to differentiate risky areas. However, these animals crossed apparently unsuitable habitat matrix during dispersal which makes them more prone to encounters with dogs, humans and predators. These findings demonstrate the risk avoidance behaviour of dispersers. Further, three cases of non-collared red panda rescue and deaths were reported to the Red Panda Network (https://www.redpandanetwork.org/) within seven months (May–November) which indicates the high stress and risk to dispersers. Of these two were found dead due to dog attack, one was rescued when found being chased by a dog, and the last one was found outside the habitat range. Three of these cases were reported during the dispersal phase (March–May). Most of the mortality causes of dispersers in other mammals are also related with anthropogenic causes, especially dogs and roads [88, 89]. Furthermore dispersing individuals are more likely to be restricted to sub-optimal habitat in part because residents will defend high quality habitat [90]. Hence, there is likely to be high mortality during dispersal [17, 90].

### Effect of disturbances and fragmentation on movement

Wildlife perceives some landscape features as risky. We observed red pandas avoiding roads by increasing their speed when in their proximity. Roads usually have reduced forest cover, lower bamboo density, higher grazing disturbance, high human traffic and dog presence [91], possibly making red pandas feel insecure whilst near such features. The camera trapping data revealed human-walking tracks had relatively less traffic volume (2.7 individuals/day) in comparison to roads (8.3 individuals/day). Predators often use such linear features thus making them ecological traps [92]. Red pandas may slow down to be more vigilant when close to human tracks and very carefully crossed the trails.

Red panda’s response to cattle stations was more conspicuous during the cub-rearing season than other seasons. Animals with offspring avoid risky areas [93] as proposed by the ecology of fear hypothesis [94]. Red pandas seemed to adopt a nocturnal pattern to cope with livestock disturbances which could be ascribed to open space, increased human presence and fear of livestock herder’s dogs [95]. Their high speed in unsuitable matrix could be their adaptation to minimize encounters with dogs and human-induced threats [95]. Likewise, for red panda small-sized habitat patches and habitat patches with low forest cover may not supply enough resources for diet, resting and security from predation risk [96]. Such small-sized habitat patches impose high movement costs [97], which may have provoked red pandas to avoid such patches by moving faster following less tortuous paths. These movement patterns provide further support for the hypothesis that animals move faster and follow less tortuous path in risky and fragmented habitat.

## Conclusions

In a world ever more dominated by human activities it is increasingly important to understand how wild animals adapt in anthropogenic landscapes. Our approach can be applied for evaluating how a species inhabiting the human-dominated landscape responds to disturbances. Notwithstanding this study is based on a single species, our findings have implications for the conservation of habitat and diet specialists. Firstly, the data show that landscape attributes and disturbances can directly influence an animal’s movement pattern. Despite the small sample size, the large dispersal distance has highlighted the potential impact of habitat fragmentation and the importance of undisturbed and continuous habitat extended over large scale for the conservation of red panda and other habitat specialists. Secondly, our study highlights the importance of habitat management during biologically critical periods, particularly during the mating, birthing, cub-rearing and dispersal. We recommend habitat zonation to limit human activities and avoid disturbances, especially livestock herding and road construction in core areas. Thirdly, we suggest improving functional connectivity by increasing inter-patch and intra-patch connectivity guided by resistance and habitat suitability analyses. Lastly, it is obvious that surviving in human-modified habitat is challenging due to the high energetic cost to adapt in such fearful landscapes. Therefore further studies should evaluate the stress level and energetic cost of animals living in such habitat. In addition, we also recommend further study on (1) the effect of disturbances and fragmentation on space-use, interaction, and resource-selection patterns; and (2) the dynamics of activity and movement patterns in response to climatic factors and anthropogenic disturbances.

## Supplementary Information

**Additional file 1: Table S1.** Summary of the minimum, maximum and mean monthly temperatures (°C) recorded in the study area from March 2017 to February 2018. **Table S2.** Models describing daily distance as a function of age, sex and season. **Table S3.** Effects of age, sex and season on daily distance. **Table S4.** Models describing step length as a function of age, sex, diel time and season. **Table S5.** Effects of age, sex, diel time and season on activity level. **Table S6.** Models describing step length as a function of disturbance variables, fragmentation metrics and season. **Figure S1.** Dendrogram of disturbance variables and fragmentation metrics. **Figure S2.a.** Frequency distribution of disturbance variables and fragmentation metrics. **Figure S2.b.** Frequency distribution of disturbance variables for randomly generated points. **Figure S3.** Time-series plots depicting the proximity distance between cubs and their mothers before and after the dispersal. **Figure S4.** Movement trajectories of dispersers showing dispersal and nondispersal phases. **Figure S5.** Dispersal paths of two sub-adult female red pandas.

## Data Availability

The datasets generated and analysed during the current study are not publicly available due to risk of poaching but are available from the corresponding author on reasonable request.

## Abbreviations

AIC: Akaike’s Information Criterion
AREA: Patch area
CLUMPY: Clumpiness Index
CONNECT: Connectance Index
ENN: Euclidean Nearest Neighbour Index
FRAC: Fractal Dimension Index
GPS: Global positioning system
IQR: Interquartile range
LMM: Linear mixed model
PD: Patch density
PLAND: Proportion of land cover
TPI: Topographic Position Index
VHF: Very high frequency
