# Supplementary material for: Movement and dispersal of a habitat specialist in human-dominated landscapes: a case study of the red panda
Source: Mov Ecol. 2021 Dec 14;9:62. doi: 10.1186/s40462-021-00297-z (PMC8670026; doi:10.1186/s40462-021-00297-z)
Supplement: Supplementary file 1 — Additional file 1: Table S1. Summary of the minimum, maximum and mean monthly temperatures (°C) recorded in the study area from March 2017 to February 2018. Table S2. Models describing daily distance as a function of age, sex and season. Table S3. Effects of age, sex and season on daily distance. Table S4. Models describing step length as a function of age, sex, diel time and season. Table S5. Effects of age, sex, diel time and season on activity level. Table S6. Models describing step length as a function of disturbance variables, fragmentation metrics and season. Figure S1. Dendrogram of disturbance variables and fragmentation metrics. Figure S2.a. Frequency distribution of disturbance variables and fragmentation metrics. Figure S2.b. Frequency distribution of disturbance variables for randomly generated points. Figure S3. Time-series plots depicting the proximity distance between cubs and their mothers before and after the dispersal. Figure S4. Movement trajectories of dispersers showing dispersal and nondispersal phases. Figure S5. Dispersal paths of two sub-adult female red pandas. [file 40462_2021_297_MOESM1_ESM.docx]

**Additional File 1**

**Table S1.** Summary of the minimum, maximum and mean monthly temperatures (°C) recorded in the study area from March 2017 to February 2018.

| Month | Mean | SD | Minimum | Maximum |
| --- | --- | --- | --- | --- |
| January | 5.81 | 4.27 | 0 | 14.50 |
| February | 4.45 | 2.89 | 0 | 9.80 |
| March | 9.48 | 6.06 | 0 | 28.00 |
| April | 15.97 | 4.52 | 9.80 | 24.20 |
| May | 17.70 | 3.64 | 10.60 | 27.60 |
| June | 18.98 | 3.46 | 13.60 | 28.90 |
| July | 18.68 | 3.57 | 13.30 | 27.90 |
| August | 17.97 | 3.47 | 12.10 | 27.10 |
| September | 16.74 | 4.73 | 9.00 | 26.90 |
| October | 11.37 | 4.79 | 3.80 | 22.30 |
| November | 9.71 | 5.65 | 1.60 | 24.60 |
| December | 9.54 | 6.82 | 1.70 | 27.60 |

**Table S2.** Models describing daily distance as a function of age, sex and season. First four top models resulting from model selection based on AIC are shown. Model with the smallest AIC was retained.

| Model | df | logLik | AIC | ΔAIC | weight |
| --- | --- | --- | --- | --- | --- |
| Age + Season + Sex + Age*Season + Sex*Season | 14 | -4180.43 | 8389.6 | 0 | 1 |
| Age + Season + Sex + Sex*Season | 11 | -4200.66 | 8423.8 | 34.19 | 0 |
| Age + Season + Sex + Age*Season | 11 | -4201.78 | 8426 | 36.42 | 0 |
| Season + Sex + Sex*Season | 10 | -4206.65 | 8433.7 | 44.08 | 0 |

**Table S3.** Effects of age (adult, subadult), sex (female, male) and season (mating, premating, cub-rearing, birthing) on daily distance. Estimates are based on model with the smallest AIC* (see Table S3). Significant variables are highlighted in bold.

| Variable*^#^* | Estimate | SE | df | t-value | *p* |
| --- | --- | --- | --- | --- | --- |
| (Intercept) | 794.89 | 91.89 | 8.64 | 8.65 | 0.00 |
| Age[Subadult] | 66.06 | 163.70 | 11.75 | 0.40 | 0.69 |
| Season[Cub-rearing] | 45.27 | 82.16 | 552.21 | 0.55 | 0.58 |
| Season[Premating] | -91.11 | 74.69 | 552.77 | -1.22 | 0.22 |
| Season[Birthing] | -143.16 | 84.90 | 554.88 | -1.69 | 0.09 |
| **Sex[Male]** | **679.14** | **152.38** | **13.11** | **4.46** | **0.00** |
| Age[Subadult]*Season[Cub-rearing] | -269.63 | 142.52 | 548.46 | -1.89 | 0.07 |
| Age[Subadult]*Season[Premating] | -280.14 | 180.33 | 554.77 | -1.55 | 0.12 |
| Age[Subadult]*Season[Birthing] | -80.82 | 144.44 | 555.30 | -0.56 | 0.57 |
| **Season**[**Cub-rearing**]***Sex**[**Male**] | **-381.53** | **141.56** | **458.97** | **-2.70** | **0.00** |
| Season[Premating]*Sex[Male] | -321.10 | 174.69 | 553.49 | -1.84 | 0.06 |
| **Season**[**Birthing**]***Sex**[**Male**] | **-321.11** | **149.06** | **450.00** | **-2.15** | **0.03** |

* Marginal *R^2^* = 0.2, Conditional *R^2^* = 0.25; *^#^* Adult, female and mating are the references for age, sex and season respectively.

**Table S4.** Models describing step length as a function of age, sex, diel time and season. First four top models resulting from model selection based on AIC are shown. Models with ΔAIC < 4 were retained for model averaging.

| Model*^#^* | df | logLik | AIC | delta | weight |
| --- | --- | --- | --- | --- | --- |
| Age + Season + Sex + Diel_time + Season*Sex + Season*Diel_time + Sex*Diel_time | 26 | -20119.2 | 40290.7 | 0 | 0.86 |
| Season + Sex + Diel_time + Season*Sex + Season*Die_ time + Sex*Diel_time | 25 | -20122.1 | 40294.5 | 3.77 | 0.13 |
| Age + Season + Sex + Diel_time + Season*Sex + Season*Diel_time | 23 | -20126.7 | 40299.6 | 8.91 | 0.01 |
| Age + Season + Sex + Diel_time + Season*Diel_time + Sex*Diel_time | 23 | -20128.4 | 40303.1 | 12.37 | 0.00 |

*^#^* Diel_time: diel time

**Table S5.** Effects of age (adult, subadult), sex (female, male), diel time (dawn, day, dusk, night) and season (mating, premating, cub-rearing, birthing) on activity level. Estimates are based on model average from the set of top models with ΔAIC < 4* (see Table S5). Significant variables are highlighted in bold.

| Variable*^#^* | Estimate | SE | df | t-value | *p* |
| --- | --- | --- | --- | --- | --- |
| (Intercept) | 36.36 | 7.33 | 12.95 | 4.96 | 0.000 |
| Age[Adult] | 2.09 | 7.19 | 6.96 | 0.29 | 0.779 |
| Season[Birthing] | 1.39 | 5.62 | 4,200.75 | 0.25 | 0.805 |
| **Season[Cub-rearing**] | **14.30** | **5.21** | **4,200.58** | **2.74** | **0.006** |
| Season[Premating] | 1.10 | 4.68 | 4,202.98 | 0.24 | 0.814 |
| Sex[Male] | 1.28 | 8.57 | 19.52 | 0.15 | 0.882 |
| Diel_time[Day] | 2.44 | 4.39 | 4,198.67 | 0.56 | 0.578 |
| Diel_time[Dusk] | -4.41 | 5.96 | 4,197.91 | -0.74 | 0.460 |
| Diel_time[Night] | 2.44 | 4.49 | 4,199.07 | 0.54 | 0.586 |
| **Season**[**Birthing**]***Sex**[**Male**] | **9.25** | **4.07** | **3,277.87** | **2.27** | **0.023** |
| **Season[Cub-rearing]*Sex[Male]** | **7.70** | **4.03** | **3,411.02** | **1.91** | **0.05** |
| Season[Premating]*Sex[Male] | 7.32 | 4.78 | 3,680.63 | 1.53 | 0.126 |
| Season[Birthing]*Diel_time[Day] | -2.73 | 5.94 | 4,200.26 | -0.46 | 0.647 |
| **Season**[**Cub-rearing**]***Diel time**[**Day]** | **-13.38** | **5.53** | **4,198.73** | **-2.42** | **0.016** |
| Season[Premating]*Diel_time[Day] | -6.29 | 5.03 | 4,198.52 | -1.25 | 0.211 |
| Season[Birthing]*Diel_time[Dusk] | -2.82 | 7.71 | 4,198.64 | -0.37 | 0.714 |
| **Season**[**Cub-rearing**]***Diel_time**[**Dusk**] | **-14.46** | **7.28** | **4,198.18** | **-1.99** | **0.047** |
| Season [Premating]* Diel_time[Dusk] | 2.81 | 6.79 | 4,197.99 | 0.41 | 0.679 |
| Season [Birthing]* Diel_time[Night] | -6.37 | 6.19 | 4,198.84 | -1.03 | 0.303 |
| **Season**[**Cub-rearing**]***Diel_time**[**Night**] | **-17.48** | **5.71** | **4,198.45** | **-3.06** | **0.002** |
| Season[Premating]*Diel_time[Night] | -5.42 | 5.12 | 4,199.81 | -1.06 | 0.289 |
| Sex[Male]*Diel_time[Day] | -5.32 | 5.23 | 4,199.76 | -1.02 | 0.308 |
| Sex[Male]*Diel_time[Dusk] | -5.84 | 6.31 | 4,201.64 | -0.93 | 0.355 |
| Sex[Male]*Diel_time[Night] | -6.00 | 5.46 | 4,204.83 | -1.10 | 0.271 |

* Marginal *R^2^* = 0.02, Conditional *R^2^* = 0.11; *^#^* Sub-adult, female, dawn and mating are the references of age, sex, time and season respectively.

**Table S6.** Models describing step length as a function of disturbance variables, fragmentation metrics and season. First 20 top models resulting from model selection based on AIC are shown. Models with ΔAIC < 4 were retained for model averaging.

| Model*^#^* | df | LogLik | AIC | ΔAIC | weight |
| --- | --- | --- | --- | --- | --- |
| Catt_dist + Season | 7 | -3190.79 | 6395.6 | 0 | 0.14 |
| AREA + Catt_dist + Season | 8 | -3189.96 | 6396 | 0.35 | 0.12 |
| Catt_dist + ENN + Season + Catt_dist*Season | 11 | -3186.99 | 6396.1 | 0.46 | 0.11 |
| Catt_dist + ENN + Trac_dist + Season + Catt_dist*Season | 12 | -3186.44 | 6397 | 1.39 | 0.07 |
| AREA + FRAC + Season | 7 | -3191.53 | 6397.1 | 1.48 | 0.07 |
| Catt_dist + FRAC + Season | 8 | -3190.93 | 6397.9 | 2.3 | 0.04 |
| Catt_dist + Trac_dist + Season | 8 | -3191.14 | 6398.3 | 2.72 | 0.04 |
| AREA + Catt_dist + Trac_dist + Season | 9 | -3190.17 | 6398.4 | 2.8 | 0.03 |
| Catt_dist + Road_dist + Trac_dist + ENN + Season + Catt_dist*Season | 13 | -3186.25 | 6398.7 | 3.04 | 0.03 |
| Diel_time + Catt_dist + ENN + Season + Diel_time*Catt_dist + Catt_dist*Season | 13 | -3186.27 | 6398.7 | 3.06 | 0.03 |
| AREA + Catt_dist + Season | 6 | -3193.45 | 6398.9 | 3.31 | 0.03 |
| AREA + Catt_dist + Road_dist + Trac_dist + Season | 8 | -3191.54 | 6399.1 | 3.51 | 0.02 |
| Catt_dist + Road_dist + Trac_dist + Season | 10 | -3189.54 | 6399.2 | 3.55 | 0.02 |
| Catt_dist + Road_dist + Season | 9 | -3190.56 | 6399.2 | 3.58 | 0.02 |
| AREA + Catt_dist + Season + Catt_dist*Season | 8 | -3191.6 | 6399.3 | 3.64 | 0.02 |
| Catt_dist + Season Catt_dist*Season | 11 | -3188.61 | 6399.3 | 3.71 | 0.02 |
| AREA + Catt_dist + Road_dist + Season | 10 | -3189.73 | 6399.6 | 3.94 | 0.02 |
| AREA + Catt_dist + ENN + Season + Catt_dist*Season | 9 | -3190.77 | 6399.6 | 3.99 | 0.02 |
| AREA + Diel_time + Catt_dist + Season + Diel_time*Catt_dist | 12 | -3187.8 | 6399.7 | 4.11 | 0.02 |
| Diel_time + Catt_dist + Trac_dist + ENN + Season + Diel_time*Catt_dist | 10 | -3189.85 | 6399.8 | 4.17 | 0.02 |

*^#^* AREA: patch area, ENN: euclidean nearest neighbour index, FRAC: fractal dimension index, Catt_dist: distance to cattle station, Trac_dist: distance to walking tracks; Road_dist: distance to road, Diel_time: diel time


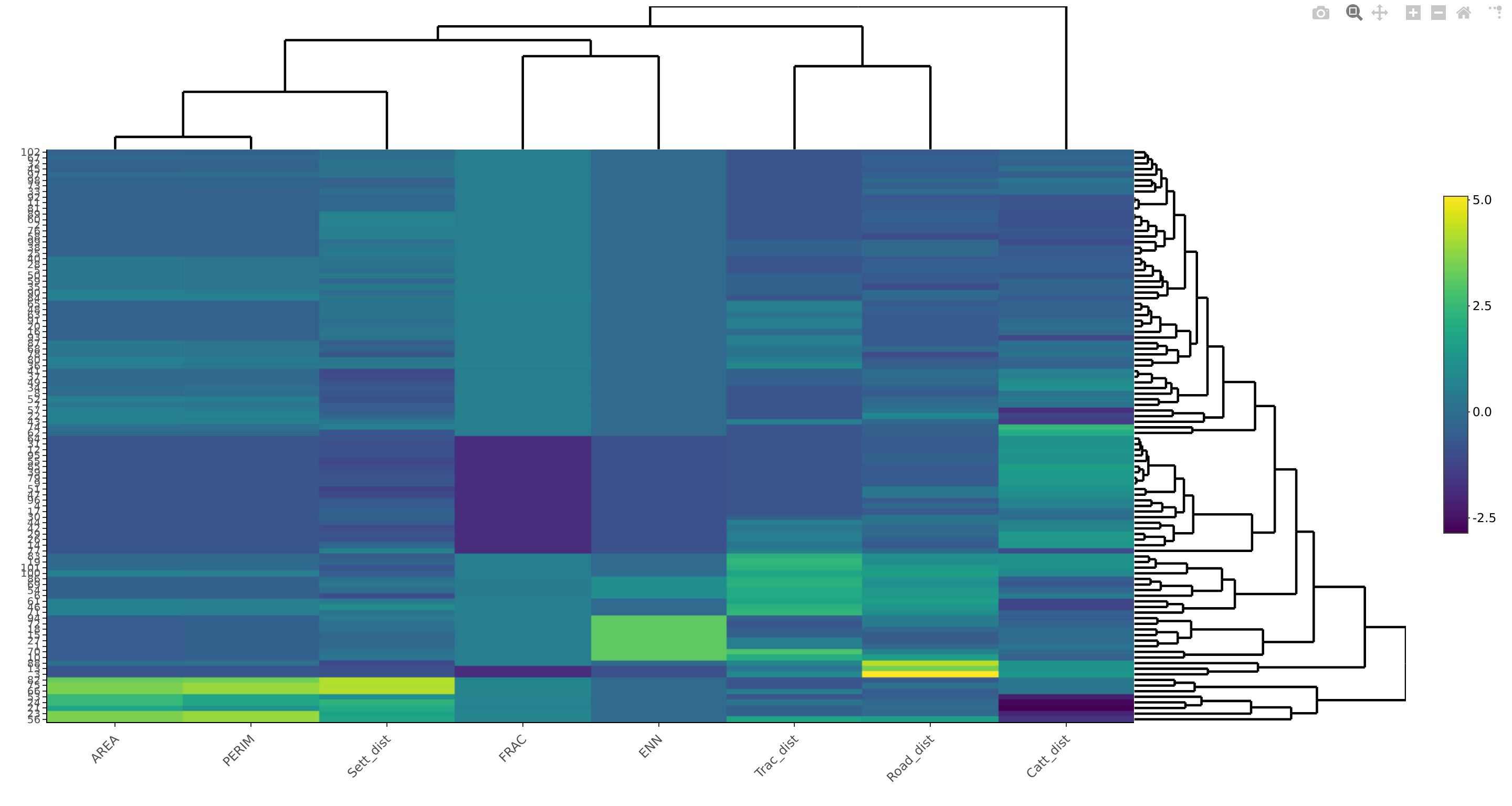


**Figure S1.** Dendrogram of disturbance variables and fragmentation metrics. This dendrogram represents 2% of randomly chosen data which shows how variables are interlinked at patch level. This is based on average hierarchical clustering method with the step length in rows and disturbances and fragmentation metrics in columns. The step length increases from violet to yellow.


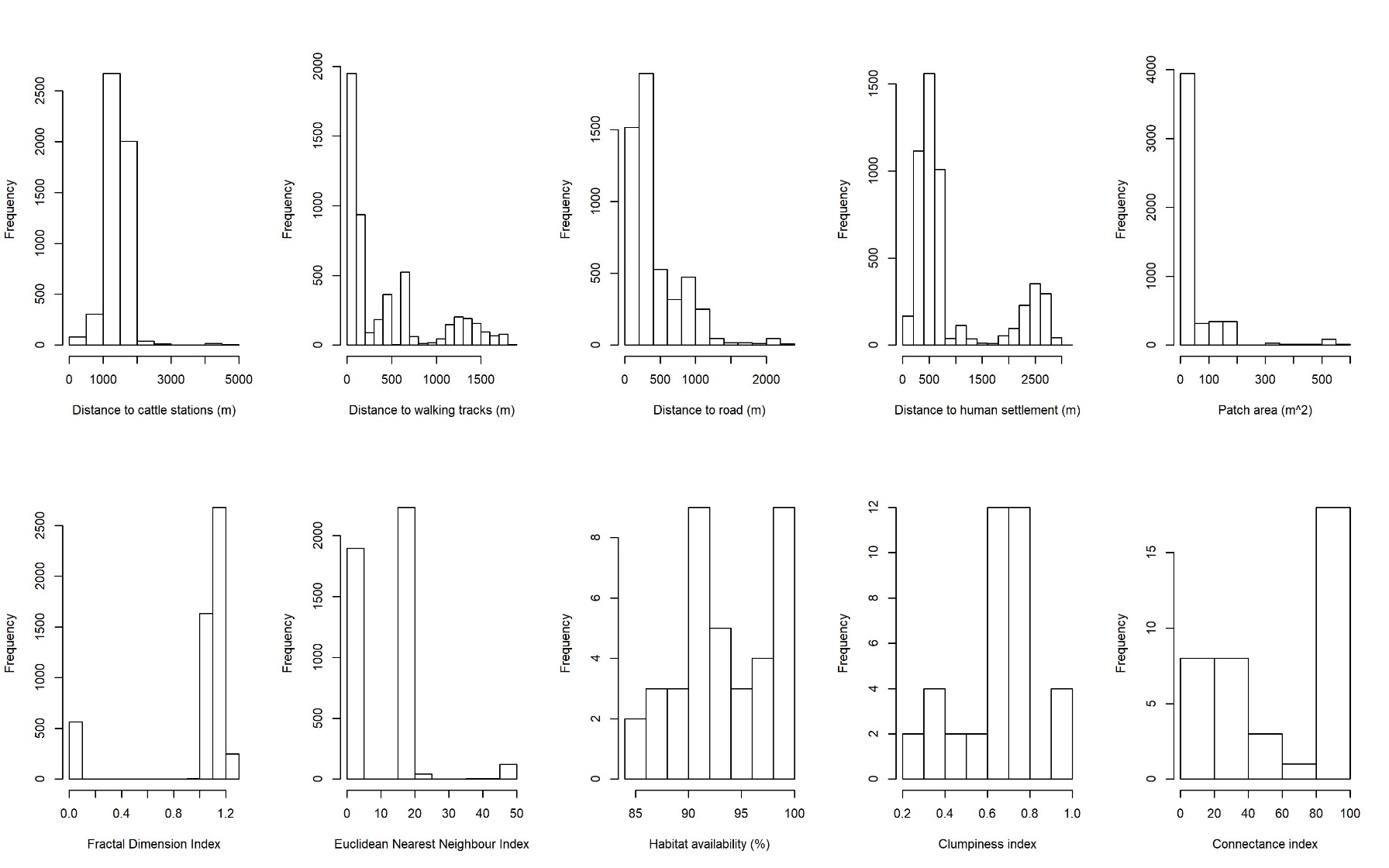


**Figure S2.a.** Frequency distribution of disturbance variables and fragmentation metrics. Shapiro-Wilk normality test showed that these variables had a non-parametric distribution (*p* < 0.0001). This pattern shows the human-dominated nature of the landscape.


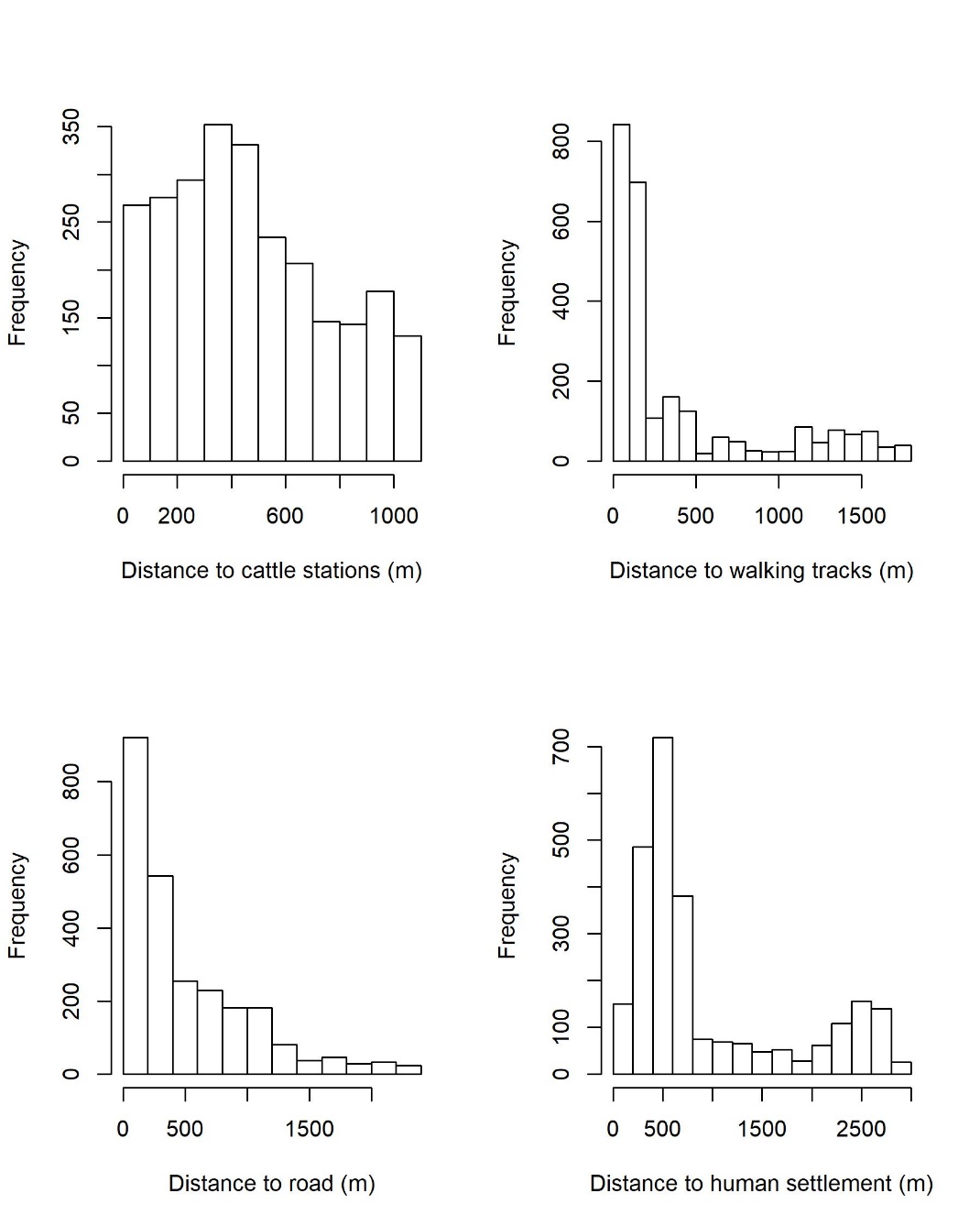


**Figure S2.b.** Frequency distribution of disturbance variables for randomly generated points. We generated equal number random points (n=5,130) of the total presence points in ArcMap V10.8. All these variables have skewed distributions, except the distance to cattle stations. However, the Shapiro Wilk normality test showed all these variables having non-parametric distributions (*p* < 0.0001), which is similar to the distribution pattern of the original presence data.


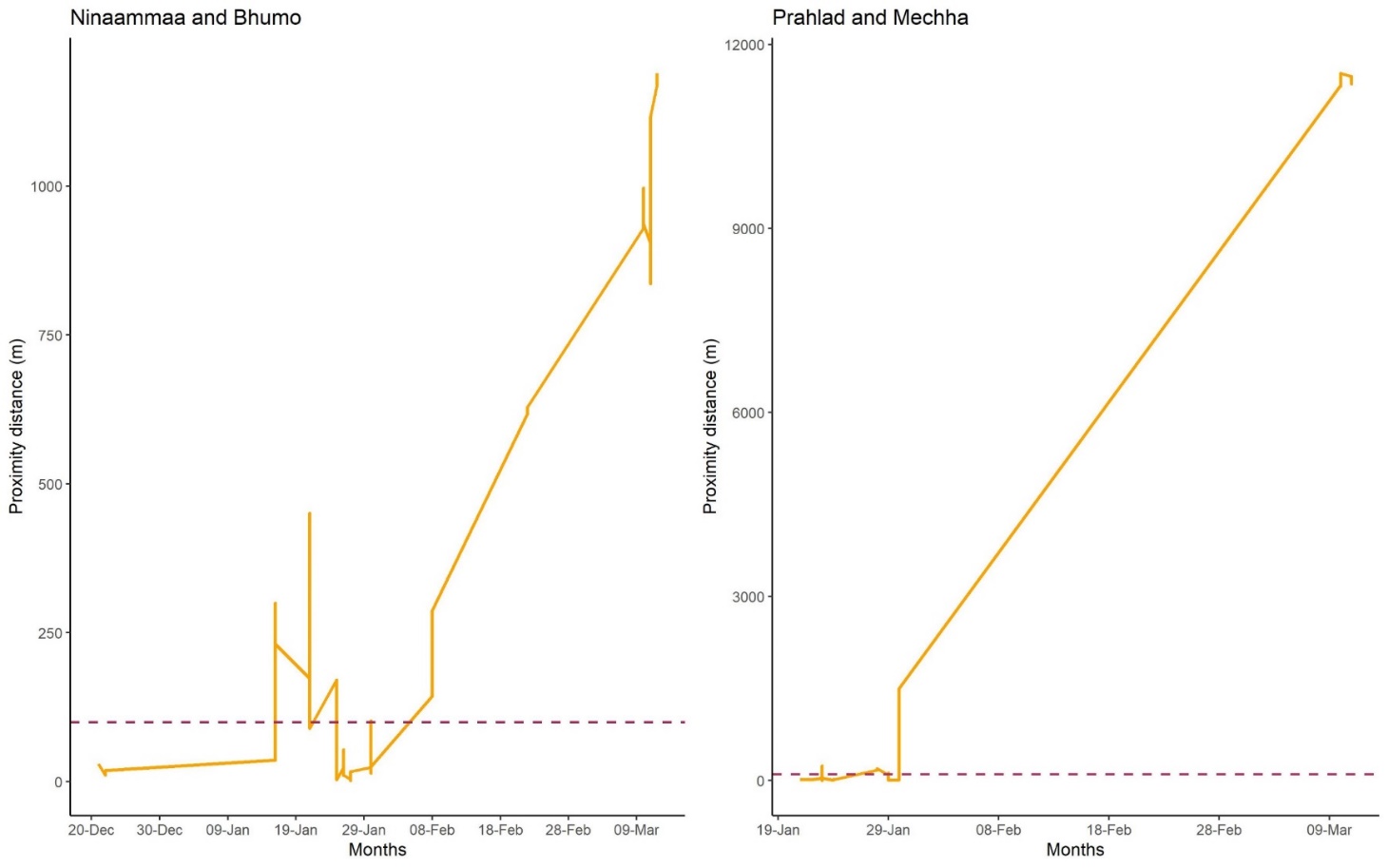


**Figure S3.** Time-series plots depicting the proximity distance between cubs and their mothers before and after the dispersal. Two pairs of mothers and cubs separated between 30 January and 7 February 2020. Their separation coincided with the onset of the new moon. But they lived in the natal area for three to five weeks before starting dispersal away from their natal range. The x-axis represents the date and y-axis shows the distance between a pair of cubs and mothers. The red line is the threshold at 100 m distance.


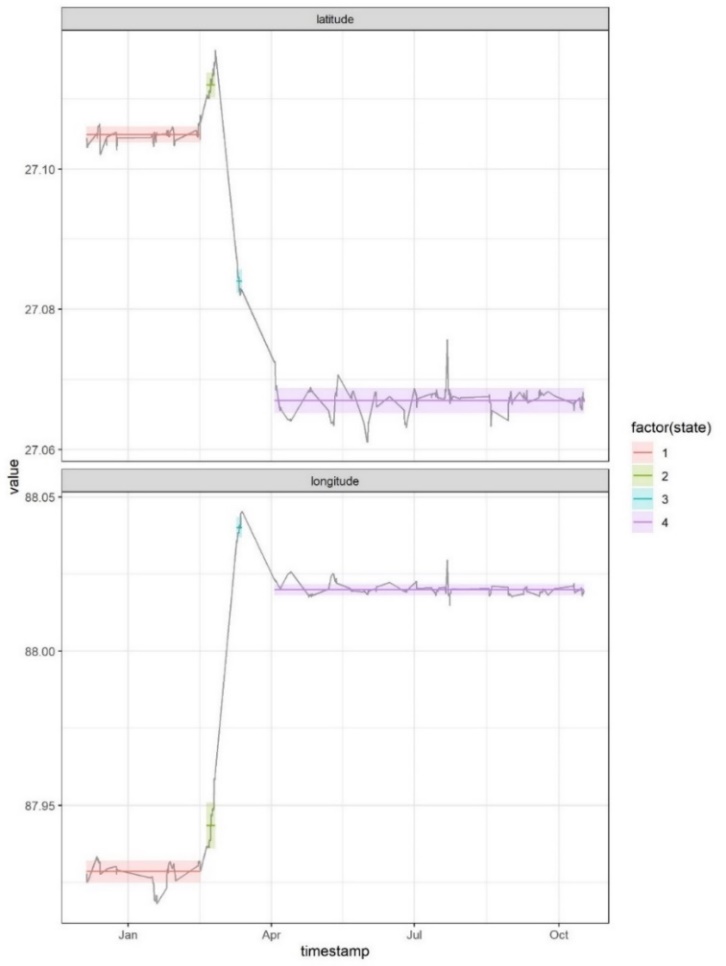

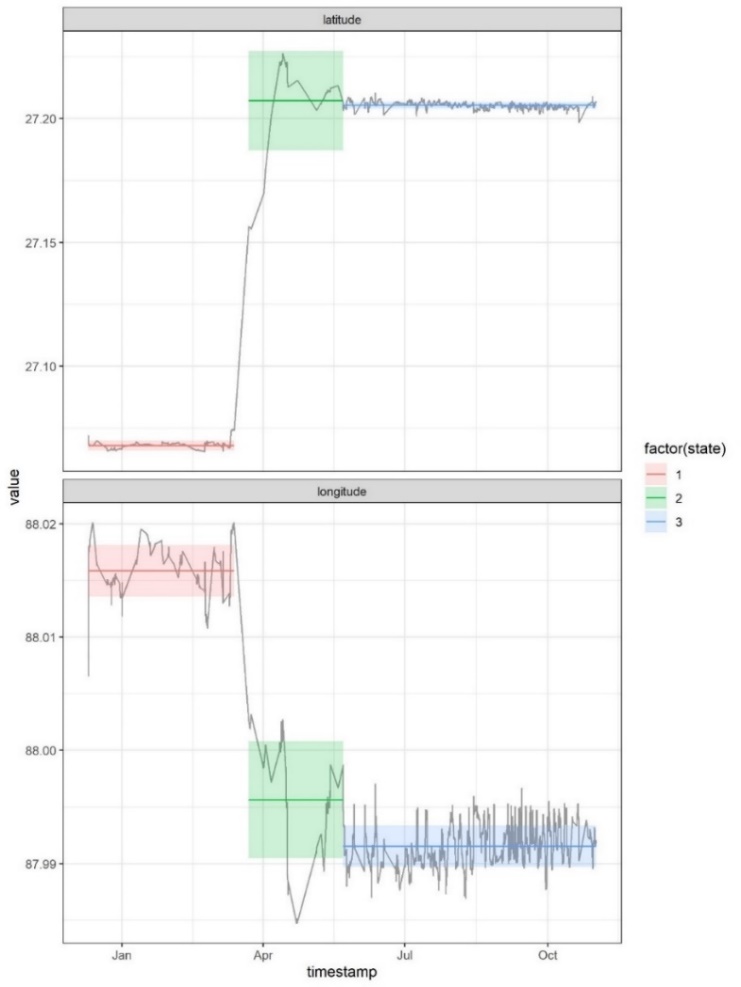


**b.**

**a.**

**Figure S4.** Fragments of dispersers showing dispersal and non-dispersal phases. The x-axis represents timestamp in months and y-axis connotes latitude (above) and longitude (below) of locations. The coloured ribbons show the 95% CI. The first and last stationary phases represent non-dispersal phases (natal home and new home). **a).** Fragmented movement trajectory of sub-adult female 1. Of the three clusters, first (red) and last (blue) clusters as non-dispersal phase while the second cluster (green) connotes the dispersal phase. **b).** Fragmented movement trajectory of sub-adult female 12. Of the four clusters, first (red) and last (purple) clusters as non-dispersal phase while the second (yellow) and third (blue) clusters connote the dispersal phase.


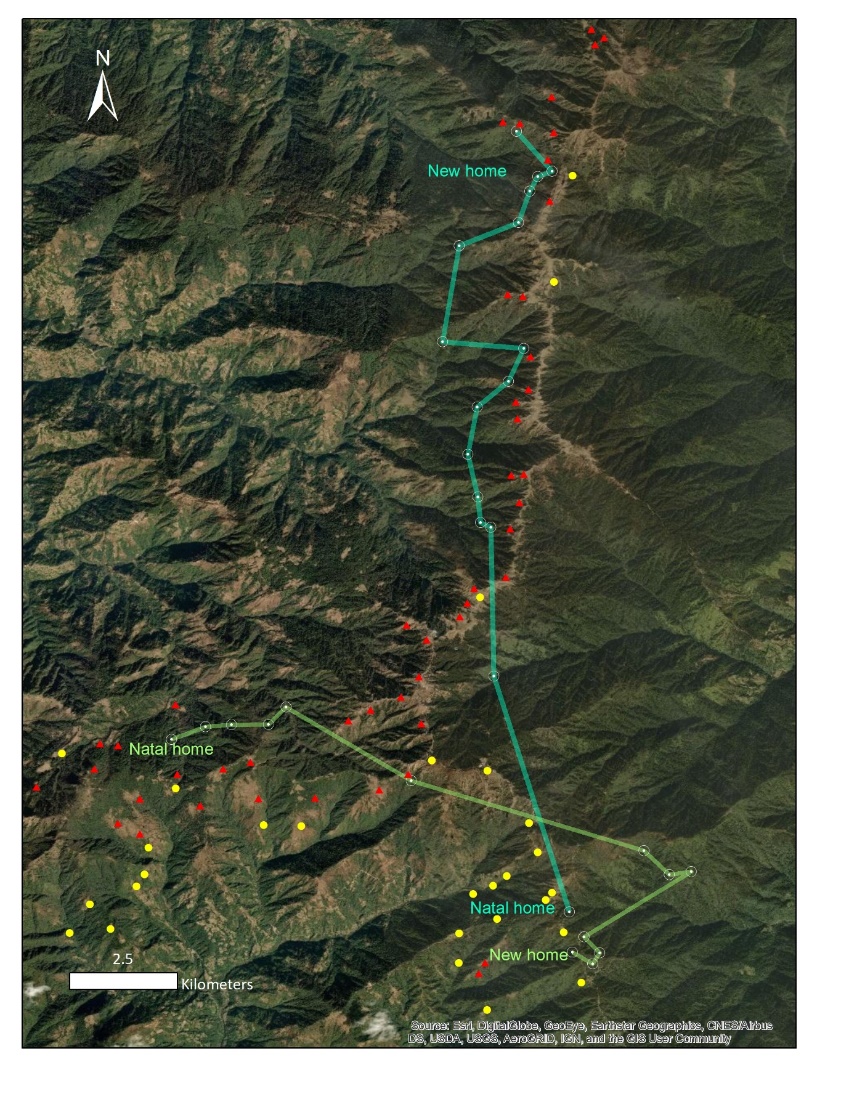


**Figure S5.** Dispersal paths of two sub-adult female red pandas. One collared sub-adult male did not disperse from its natal site while two sub-adult females travelled 17.95 and 24.1 km respectively. The blue and light-yellow lines represent the dispersal path followed by these two females. White dots on these lines show locations recorded during dispersal. Forest cover area is shown in green while brown area represents degraded forest and non-forest area. Habitations and cattle stations are shown in red and yellow dots respectively.
